# *Babesia vesperuginis*, a neglected piroplasmid: new host and geographical records, and phylogenetic relations

**DOI:** 10.1186/s13071-017-2536-3

**Published:** 2017-12-06

**Authors:** Alexandra Corduneanu, Kristýna Hrazdilová, Attila D. Sándor, Ioana Adriana Matei, Angela Monica Ionică, Levente Barti, Marius-Alexandru Ciocănău, Dragoş Ștefan Măntoiu, Ioan Coroiu, Sándor Hornok, Hans-Peter Fuehrer, Natascha Leitner, Zoltán Bagó, Katharina Stefke, David Modrý, Andrei Daniel Mihalca

**Affiliations:** 1 Department of Parasitology and Parasitic Diseases, University of Agricultural Sciences and Veterinary Medicine of Cluj-Napoca, Cluj-Napoca, Romania; 2 CEITEC VFU, University of Veterinary and Pharmaceutical Sciences, Brno, Czech Republic; 3 Romanian Bat Protection Association- Central Branch, Odorheiu Secuiesc, Romania; 4 Department of Infection Diseases, University of Agronomical Sciences and Veterinary Medicine, Bucharest, Romania; 5 Institute of Speleology ‘Emil Racoviţă’, Cluj-Napoca, Romania; 6 Faculty of Biology and Geology, University Babes- Bolyai, Cluj-Napoca, Romania; 7 Department of Parasitology and Zoology, University of Veterinary Medicine, Budapest, Hungary; 8 Institute of Parasitology, Department of Pathobiology, University of Veterinary Medicine, Vienna, Austria; 9 Institute for Veterinary Disease Control, Austrian Agency for Health and Food Safety (AGES), Robert Koch Gasse 17, 2340 Mödling, Austria; 10 Museum of Natural History, Vienna, Austria; 11 Department of Pathology and Parasitology, University of Veterinary and Pharmaceutical Sciences, Brno, Czech Republic; 12 Biology Centre, Institute of Parasitology, Czech Academy of Sciences, České Budějovice, Czech Republic; 13 Department of Virology, Veterinary Research Institute, Hudcova 296/70, 621 00 Brno, Czech Republic

**Keywords:** Bats, Tick-borne pathogens, Piroplasms, *Babesia vesperuginis*, Europe

## Abstract

**Background:**

*Babesia* spp. are hemoparasites which infect the red blood cells of a large variety of mammals. In bats, the only known species of the genus is *Babesia vesperuginis*. However, except a few old reports, the host range and geographical distribution of this bat parasite have been poorly studied. This study aimed to investigate the presence of piroplasms in tissues of bats collected in four different countries from eastern and central Europe: Austria, Czech Republic, Hungary and Romania.

**Methods:**

A total of 461 bat carcasses (24 species) were collected between 2001 and 2016 from caves, mines and buildings. PCR was performed using specific primers targeting a portion of the 18S rDNA nuclear gene and cytochrome *c* oxidase subunit 1 mitochondrial gene, followed by sequencing.

**Results:**

The results of this study show for the first time the presence of *B. vesperuginis* in bats in central and eastern Europe. The phylogenetic analysis of the 18S rDNA nuclear gene revealed no variability between the sequences and the phylogenetic analysis of the *cox*1 mitochondrial gene proved that *B. vesperuginis* could be divided into two subclades.

**Conclusion:**

Our study showed a broad geographical distribution of *B. vesperuginis* in European bats, reporting its presence in five new host species (*M.* cf. *alcathoe*, *M. bechsteinii*, *M. myotis*, *Pi. nathusii* and *V. murinus*) and three new countries.

**Electronic supplementary material:**

The online version of this article (10.1186/s13071-017-2536-3) contains supplementary material, which is available to authorized users.

## Background

Chiroptera is the second largest order of mammals and includes about 20% of all mammal species worldwide [1]. Studies on the epidemiological role of chiropterans in the transmission of pathogens have focused mainly on zoonotic viruses such as rabies [2, 3], acute respiratory syndrome (SARS) [4], Ebola [5], Zika [6], and other viral disease (influenza, acute respiratory illness, chikungunya) [7]. Compared with other mammals, the role of bats in the transmission cycle of tick-borne protists [8, 9] and bacteria are less studied [10, 11]. The life-cycle of most of the *Babesia* spp. in domestic animals is well known and involves a hard tick as a definitive host [12]. However, for bat piroplasms, the life-cycle (including a complete range of the vertebrate hosts) and the vectors involved are unknown.

*Babesia vesperuginis* was described by Dionisi [13] from *Nyctalus noctula* in Italy and later found also in *Pipistrellus* sp. in Italy [14]. The species was later reported in the UK [15] in blood smears of bats, followed by experimental transmission studies [16]. Concannon et al. [17] identified the infection with *B. vesperuginis* by PCR targeting the 18S rDNA in six individuals from a total of 60 bats from Cornwall, UK, and they concluded that the parasite is different from other known *Babesia*. The only study outside Europe reports the presence of unidentified *Babesia* in *Mormoops megalophylla* from Colombia, with a low microscopic prevalence of 1.19% in blood smears [18]. In general, the diversity and ecology of bat piroplasmids remains unknown, and there is no data regarding how the parasite is transmitted. Hornok et al. [8] studied the presence of apicomplexan protozoans in bat faeces from Hungary and Romania. All samples were tested for the presence of piroplasms DNA with a conventional PCR and the positive samples (2.25%) have shown similarity with *Babesia canis*.

The aim of this study was to investigate the presence of piroplasmids and their genetic diversity in bats from central and eastern Europe, namely from Austria, Czech Republic, Hungary and Romania based on partial sequences of nuclear 18S rRNA and mitochondrial *cox*1 genes to broaden the knowledge on their host spectrum, geographical distribution and phylogenetic relationships to other piroplasms.

## Methods

**Fig. 1:**
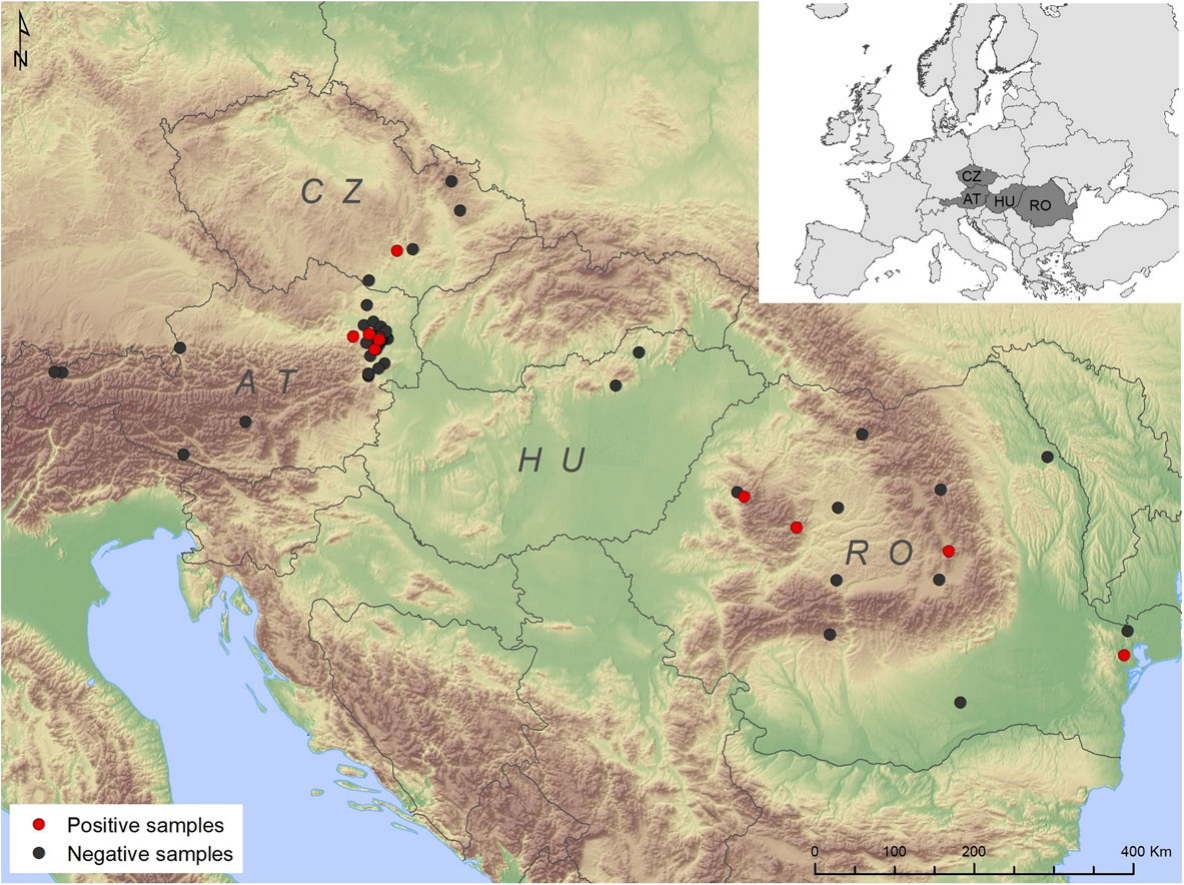
Sampling sites: red dots represent the positive locations for *Babesia vesperuginis*; black dots represent the negative locations (including multiple samples from the same place). *Abbreviations*: AT, Austria; CZ, Czech Republic; HU, Hungary; RO, Romania

Heart tissue from 461 bats collected in four different countries (Austria, Czech Republic, Hungary and Romania) between 2001 and 2016 were examined (Additional file 1: Table S1, Fig. 1). All animals were found either as accidental kills of wind power generators, dead due to natural causes or euthanized because of progressive deterioration of general condition (in few captive specimens). A wind farm in Babadag, Romania, consisting of 20 turbines was monitored for a period of four years (2013–2016) using a weekly time frame with two consecutive days of carcass searches, from April to November. Bat carcases were found either fresh or desiccated. Samples were collected from carcasses which have been labelled fresh. These have been found on the second day of each weekly field visit and presented no signs of maggots or decomposition. All bats were identified according to morphological keys [19] and stored in 96% ethanol, at -80 °C (samples from Austria) or in a freezer until their necropsy. Morphological identification of whiskered bats from the *Myotis mystacinus* group (*M. alcathoe*, *M. brandtii* and *M. mystacinus*) is not only problematic, but these species may show signs of hybridization [20]. Therefore, we distinguished these as the ‘most likely’ morphological species (e.g. *Myotis* cf. *alcathoe* in case of a bat identified morphologically as *M. alcathoe*). Genomic DNA was extracted from 25 mg of heart tissue using DNeasy Blood & Tissue Kit (Qiagen, Hilden, Germany), according to the manufacturer’s instruction and stored at -20 °C.

A nested PCR targeting a 561 bp fragment of 18S rDNA using previously described primers [21, 22] was used for initial screening. The reactions were carried out in a 25 μl reaction mixture containing 12.5 μl 2× Green PCR Master Mix (Rovalab GmBH, Teltow, Germany), 5.5 μl water, 1 μl of each primer (10 pmol/ μl) and 5 μl aliquot of isolated DNA in the first round and in the second round instead of DNA 2 μl of PCR product from the first round was used. The PCR was performed using the T1000™ Thermal Cycler (Bio-Rad, London, UK) with the following condition: initial denaturation at 95 °C for 3 min, then 40 cycles of denaturation at 95 °C for 30 s, annealing at 60 °C for 30 s (for the first round), 50 °C for 30 s (for the second round) and extension at 72 °C for 1 min (for the first round), 72 °C for 40 s (for the second round) and a final extension at 72 °C for 7 min. For each set of reactions (45 samples) 2 negative controls (distilled water) and one positive control which was DNA isolated from the blood of a naturally infected dog with *Babesia canis* were included.

For the samples positive for 18S rDNA, an additional nPCR targeting the *cox*1 gene was applied using a modified protocol described by Gou et al. [23] with the following primers Bab_For1: (5′-ATW GGA TTY TAT ATG AGT AT-3′), Bab_Rev1: (5′-ATA ATC WGG WAT YCT CCT TGG-3′) for the first round and Bab_For2: (5′-TCT CTW CAT GGW TTA ATT ATG ATA T-3′), Bab_Rev2: (5′-TAG CTC CAA TTG AHA RWA CAA AGT G-3′) for the second round. The amplification was performed as follows: 25 μl reaction mixture containing 2 μl aliquot of isolated DNA in the first round and 1 μl in the second, 12.5 μl Master Mix (PCRBIO *Taq* Mix Red), 1 μl of each primer (10 pmol/μl) and 8.5 μl water. The amplification profile consisted of 1 min of initial denaturation at 95 °C, followed by 35 cycles of denaturation at 95 °C for 15 s, annealing at 45 °C for 30 s (for the first round), 49 °C for 30 s (for the second round) and extension at 72 °C for 1 min, and a final extension at 72 °C for 10 min.

Amplification products were visualized by electrophoresis on 1.5% agarose gel stained with RedSafe™ 20,000× Nucleic Acid Staining Solution (Chembio, St Albans, UK), and their molecular weight was assessed by comparison to a molecular marker (O’GeneRuler ™ 100 bp DNA Ladder, Thermo Fisher Scientific Inc., Waltham, MA, USA). PCR products were purified using the QIAquick PCR purification kit (Qiagen, Hilden, Germany) and sent for sequencing (Macrogen Europe, Amsterdam, Netherlands).

The sequences were compared with those available in GenBank™ using Basic Local Alignments Tool (BLAST) analyses. All sequences were analysed and edited using Geneious® 9.1.2 software [24]. Alignments of non-coding (18S rDNA) sequences were generated using the ClustalW algorithm [25]. For coding *cox*1 sequences, translational alignment (nucleotide sequences are translated into protein, the alignment was performed on the protein sequence, and then translated back to nucleotide sequence) implemented in Geneious ® 9.1.2 using ClustalW algorithm was performed. The evolution model for each dataset was chosen based on likelihood ratio test computed by R software (R Core Team, 2012). Phylogenetic analyses were performed using the maximum likelihood method in PhyML 3.0 software [26]. Phylogenetic trees were visualized and edited in FigTree v1.4.1 (http://tree.bio.ed.ac.uk/software/figtree/).

Statistical analysis was performed using EpiInfo™ 7 software (CDC, USA). The overall prevalence of *B. vesperuginis*, the prevalence at locality level and the prevalence of each bat species and their 95% confidence interval (95% CI) were calculated. The map was generated using ArcGIS 10.3 software (Fig. 1).

## Results

PCR targeting 18S rDNA revealed the presence of piroplasmid DNA in 20 out of 461 bats (4.34%, 95% CI: 2.83–6.61). The positive samples originated from 9 different locations from three different countries, belonging to seven bat species (Tables 1 and 2). *Babesia vesperuginis* was present in *Myotis* cf. *alcathoe* (1/12), *M. bechsteinii* (1/4), *M. myotis* (1/6), *N. noctula* (4/246), *P. nathusii* (3/28), *P. pipistrellus* (6/71) and *Vespertilio murinus* (4/23). The following species were negative (numbers of examined specimens in parentheses): *Barbastella barbastellus* (*n* = 2), *Eptesicus nilssonii* (*n* = 1), *E. serotinus* (*n* = 6), *Hypsugo savii* (*n* = 11), *Miniopterus schreibersii* (*n* = 4), *M.* cf. *brandtii* (*n* = 3), *M. daubentonii* (*n* = 1), *M.* cf. *mystacinus* (*n* = 4), *M. nattereri* (*n* = 1), *N. leisleri* (*n* = 5), *Pi. kuhlii* (*n* = 8), *Pi. pygmaeus* (*n* = 5), *Pl. auritus* (*n* = 8), *Pl. austriacus* (*n* = 1), *Rhinolophus euryale* (*n* = 9), *R. ferrumequinum* (*n* = 1) and *R. hipposideros* (*n* = 1).

**Table 1:** Prevalence (%) and frequency (in parentheses) of *Babesia vesperuginis* in the positive bat species in each locality

Locality	Austria				Czech Republic			Romania	
Species	Mauerbach	Mödling	Neulengbach	Vienna	Brno	Babadag	Huda lui Papară	Muntele Puciosu	Peşterea cu apă din Valea Leşului
*Myotis alcathoe*	–	–	–	–	–	–	–	8.33 (1/12)	–
*Myotis bechsteinii*	–	–	–	–	–	–	–	25 (1/4)^a^	–
*Myotis myotis*	–	–	–	–	–	–	–	–	100 (1/1)^a^
*Nyctalus noctula*	–	–	–	–	9.09 (1/11)	8.33 (1/12)	18.18 (2/11)	–	–
*Pipistrellus nathusii*	–	–	–	–	–	12 (3/25)	–	–	–
*Pipistrellus pipistrellus*	100 (1/1)^a^	–	100 (1/1)	–	6.66 (1/15)	–	5.66 (3/53)	–	–
*Vespertilio murinus*	100 (1/1)	100 (1/1)	–	9.09 (1/11)	–	–	–	50 (1/2)	–

^a^Samples negative for the *cox*1 gene

**Table 2:** Samples from the phylogenetic analysis of 18S rRNA nuclear gene (GenBank Accesion numbers provided) and *cox*1 mitochondrial gene

Abbreviation	Species	Location	GenBank ID
Bat 1	*Nyctalus noctula*	Brno (CZ)	MG011454
Bat 2	*Nyctalus noctula*	Brno(CZ)	MG011455
Bat 4	*Vespertilio murinus*	Vienna (AT)	MG011456
Bat 5	*Vespertilio murinus*	Mödling AT)	MG011457
Bat 6	*Vespertilio murinus*	Mauerbach (AT)	MG011458
Bat 7	*Pipistrellus pipistrellus*	Neulengbach (AT)	MG011459
Bat 8	*Pipistrellus pipistrellus*	Mauerbach (AT)	MG011460
Bat 9	*Vespertilio murinus*	Muntele Puciosu (RO)	MG011461
Bat 10	*Myotis alcathoe*	Muntele Puciosu (RO)	MG011462
Bat 11	*Myotis bechsteinii*	Muntele Puciosu (RO)	MG011463
Bat 12	*Myotis myotis*	Peşterea cu apă din Valea Leşului (RO)	MG011464
Bat 13	*Nyctalus noctula*	Huda lui Papară (RO)	MG011465
Bat 14	*Nyctalus noctula*	Muntele Puciosu (RO)	MG011466
Bat 15	*Vespertilio murinus*	Mauerbach (AT)	MG011467
Bat 16	*Pipistrellus nathusii*	Babadag (RO)	MG011468
Bat 17	*Pipistrellus nathusii*	Babadag (RO)	MG011469
Bat 18	*Nyctalus noctula*	Babadag (RO)	MG011470
Bat 19	*Pipistrellus nathusii*	Babadag (RO)	MG011471
Bat 20	*Pipistrellus pipistrellus*	Huda lui Papară (RO)	MG011472
Bat 21	*Pipistrellus pipistrellus*	Huda lui Papară (RO)	MG011473

*Abbreviations*: *AT* Austria, *CZ* Czech Republic, *HU* Hungary, *RO* Romania

**Fig. 2:**
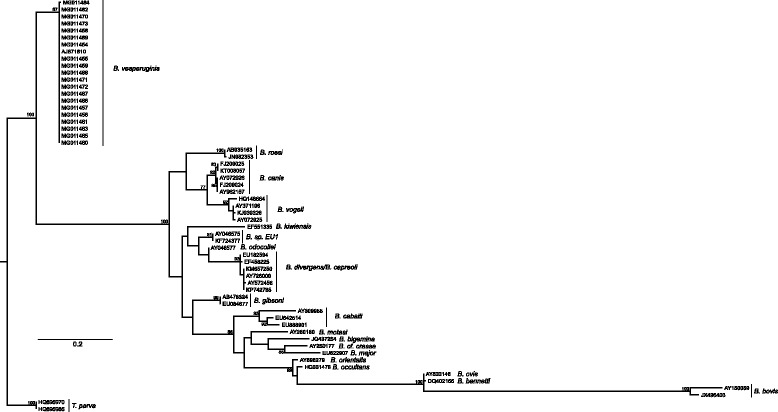
Phylogenetic tree constructed by maximum likelihood method on nucleotide sequences of 18S rRNA gene (fragment of 515 nt) of piroplasmid clade VI according to Schnittger et al. [27]. Details for sequences generated in the present study (host species and country of sample origin) are provided in Table 2. Proportion from 1000 replicates of bootsrap values only above 75% are displayed. *Theileria parva* sequences were used as the outgroup

BLAST analysis of the 18S rDNA sequences from the 20 positive samples showed a 96 to 100% similarity to *B. vesperuginis* (GenBank: AJ871610.1) isolated from *Pipistrellus* sp. in the UK. All sequences obtained from bat tissues were highly similar, except a single one from a *M. myotis* sample (GenBank: MG011464) (Peştera cu Apă din Valea Leşului, Romania), which differed by two nucleotides (Fig. 2). All sequences were submitted to the GenBank database under the accession numbers MG011454–MG011473.

**Fig. 3:**
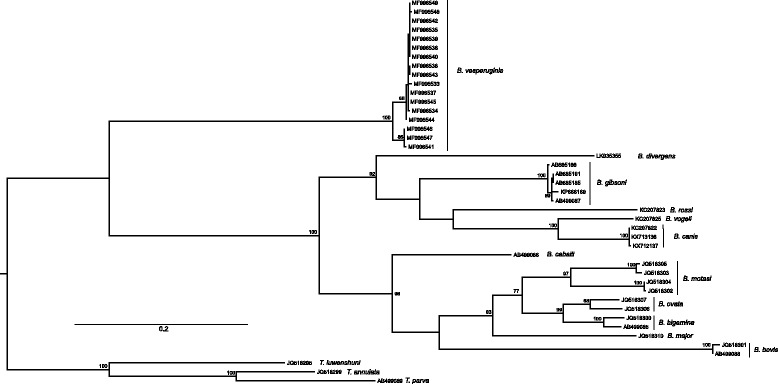
Phylogenetic tree constructed by maximum likelihood method on translational alignment of nucleotide sequence of coding region of *cox*1 gene (fragment 861 nt) of piroplasmid clade VI according to Schnittger et al. [27]. Details for sequences generated in the present study (host species and country of sample origin) are provided in Table 2. Proportion from 1000 replicates of bootsrap values only above 75% are displayed. *Theileria* spp. sequences were used as the outgroup

Additional *cox*1 PCR applied to all 18S rDNA positive samples showed a lower success of amplification (17/20). No *cox*1 sequences were available from *B. vesperuginis* in GenBank for comparative analysis. The BLAST analysis of all 17 *cox*1 sequences showed maximum 78% similarity with different isolates of *Babesia* and *Theileria*. Based on these data, a broad phylogenetic analysis including also the most related *Theileria* spp. *cox*1 sequences (clade no. V, according to Schnittger et al. [27]) was performed to confirm the phylogenetic relationships of *B. vesperuginis* with a broader range of piroplasmids (data not shown in our tree). Our *B. vesperuginis cox*1 sequences remained in a basal position within the *Babesia* clade VI, thus confirming the 18S rDNA based phylogeny. From the 17 *cox*1 sequences, 14 were similar amongst each other, forming a subclade with identity above 99.65% (maximum difference of 3 nt within 864 nt used for the phylogeny) and three of them forming a separate subclade of almost identical sequences (1 nt difference in sequence with the accession number MF996541). The subclades differ by 15–19 nt (within 861 nt fragments) among each other (Fig. 3). All sequences were submitted to the GenBank database under the accession numbers: MF996533–MF996549.

## Discussion

The samples collected for the present study originated from 24 bat species from three families including the Miniopteridae, Rhinolophidae and Vespertilionidae. All the positive animals belonged to five different genera of the Vespertilionidae. As the number of examined specimens from the other two families was low, we do not feel confident in establishing or refuting their host status for *B. vesperuginis*. Except for *N. noctula* and *Pi. pipistrellus* [13–17], all other bat species (*M.* cf. *alcathoe*, *M. bechsteinii*, *M. myotis*, *Pi. nathusii* and *V. murinus*) are new host records for *B. vesperuginis*. Our study shows for the first time the presence of *B. vesperuginis* in tissues of bats from Austria, Czech Republic and Romania.

Hornok et al. [8] found *Babesia* spp. in faeces of insectivorous bats and suggested as a likely way of infection the food ingested by bats [8]. All positive bat species forage over a range of habitats including deciduous forests, woodland edge, wetland, pasture [19]. The food of most of the positive species consists of small insects like moths [28, 29], mosquitoes [30, 31] and small dipterans [19]. The prey is caught during flight (*M. alcathoe*, *Pi. nathusii*, *Pi. pipistrellus*, *V. murinus*) or picked up from various surfaces (*M*. *bechsteinii*). There are two exceptions: *N. noctula* feeds on medium sized insects (dipterans, beetles, caddis flies) during flight and *M. myotis* feeds from the ground, on beetles, large moths, crickets and spiders [19]. By selecting food in such varied habitat types, bats may encounter the (yet) unknown vector for *B. vesperuginis*.

Another hypothesis regarding the vector of *B. vesperuginis* was presented by Gardner et al. [17] suggesting that a bat specific soft tick (*Argas vespertilionis*) may be the vector for this piroplasm species. While we did not find any soft ticks on the bats analysed, these animals might have been parasitized before at their roosts. As only larvae of soft ticks spend longer time on their hosts, their presence is hard to be detected [32, 33].

The roosting sites of sampled species are in tree hollows, buildings, cracks in cliffs or caves for the summer and underground habitats, caves for the winter except *V. murinus* which hibernates in rock fissures and crevices in tall buildings [19]. All species usually form mixed colonies with congeneric species (e.g. *M*. *bechsteinii* with *M. daubentonii*). In other cases, roosts may contain mixed colonies, with species from different genera (e.g. *M. myotis* with *Rhinolophus* spp.). In hibernating sites, even species which roost in trees may encounter a wide variety of ectoparasites, including soft ticks. Some species are sedentary (*M. alcathoe*, *M. bechsteinii*, *Pi. pipistrellus*) [19], others are adapted to migration over a few hundred kilometres (*M. myotis*) [19] and others migrate for long-distance, up to 2000 km (*N. noctula*, *Pi. nathusii*, *V. murinus*) [19, 34]. Long distance migrants feed on the go, fuelling their energy loss while migrating [34]. The range of species studied and their diverse ecology showed that *B. vesperuginis* has a wide geographical distribution among different bat populations; it can be spread over a long distance and has low bat host specificity. Most of the bat species that were negative for the presence of *B. vesperuginis* are sedentary, except *Mi. schreibersii* and *N. leisleri*. However, in most of the cases, the negativity of certain bat species for *B. vesperuginis* might have been a consequence of the small sample size.

Gardner et al. [15] found *B. vesperuginis* in two species: *Pi. pipistrellus* (19/206, 9.22%) and *M. mystacinus* (1/11, 9.09%) in UK. Concannon et al. [17] examined by PCR (targeting 18S rDNA) the heart tissue of bats and found *B. vesperuginis* only in *Pipistrellus* sp. (6/60, 10%) in the UK. All records of *B. vesperuginis*, including the present study, indicate that the main host species for *B. vesperuginis* in Europe are *N. noctula* and *Pipistrellus* spp.

The phylogenetic analysis of the 20 18S rDNA sequences showed no variability between them. However, when the more variable *cox*1 gene was used, the phylogeny demonstrated the presence of a widely distributed clade (five host species, eight localities from Austria, Czech Republic and Romania) and a smaller one, with two host species (*N. noctula* and *Pi. nathusii*). In two localities (Babadag and Huda lui Papară, Romania), sequences included in both subclades of *the cox*1 tree were present.

The life-cycle of *B. vesperuginis* is unknown. Gardener et al. [15] suggested the involvement of *Argas vespertilionis* as a vector, as this soft tick was found on the majority of the *Pi. pipistrellus* bats infected with *B. vesperuginis*. Similarly, only soft ticks (*Ornithodoros marinkellei* and *Antricola mexicanus*) were found on *Mormoops megalophylla* bats infected with *Babesia* sp. in Colombia [19]. However, the presence of *B. vesperuginis* has never been tested in soft ticks of bats. Moreover, for nearly all the *Babesia* species with a known life-cycle, the vector is a hard tick [35], suggesting a close co-evolution of piroplasms with the family Ixodidae. Nevertheless, *Babesia meri* is transmitted by *Ornithodoros erraticus* to the fat sand rat (*Psammomys obesus*) [36]. In addition, circumstantial evidence indicates the possible role of *O. moubata* (Argasidae) in the transmission of *B. gibsoni* in dogs after being artificially infected with this parasite [37], showing that the involvement of a soft tick in the life-cycle of *Babesia* sp. is possible. All hard ticks identified (a few individuals) on the individual bats included in the present study were examined and proved negative for the presence of *B. vesperuginis* in a different study [9].

## Conclusion

Our study showed a broad geographical distribution of *B. vesperuginis* in European bats, reporting its presence in five new host species (*My.* cf. *alcathoe*, *My. bechsteinii*, *My. myotis*, *Pi. nathusii*, *V. murinus*). The low variability of 18S rDNA and *cox*1 sequences and a large number of confirmed host species suggest low host specificity of this piroplasmid and imply the involvement of a rather ubiquitous vector.

## Additional file

Additional file 1:
**Table S1.** Samples distribution according to species and locality. (XLSX 15 kb)
